# Assessment of the placental microbiota of preterm infants with pneumonia: a case control study

**DOI:** 10.3389/fcimb.2025.1511141

**Published:** 2025-04-03

**Authors:** Lei Zhang, Zijin Chen, Shuai Hu, Hong Liu, Fan Lai, Yinghong Fan, Min Li, Ling Zhou

**Affiliations:** ^1^ Department of Pediatric Pulmonology, The Affiliated Chengdu Women’s and Children’s Central Hospital, School of Medicine, University of Electronic Science and Technology of China, Chengdu, Sichuan, China; ^2^ Department of Operating Room, The Affiliated Chengdu Women’s and Children’s Central Hospital, School of Medicine, University of Electronic Science and Technology of China, Chengdu, Sichuan, China; ^3^ Obstetrics and Gynecology Department, The Affiliated Chengdu Women’s and Children’s Central Hospital, School of Medicine, University of Electronic Science and Technology of China, Chengdu, Sichuan, China; ^4^ Department of Neonatology, The Affiliated Chengdu Women’s and Children’s Central Hospital, School of Medicine, University of Electronic Science and Technology of China, Chengdu, Sichuan, China

**Keywords:** placental, microbiota, preterm infants, pneumonia, 16S rRNA

## Abstract

**Objective:**

To investigate the specific characteristics and differences in the placental microbiome of preterm infants with and without pneumonia.

**Methods:**

Fifty-nine infants born at 32–36 weeks’ gestation were enrolled in this study. Among them, 33 developed pneumonia within 48 hours of birth, while the remaining 26 did not. Placental swabs were collected at birth for DNA extraction, from which the placental microbial composition was analyzed using a bioinformatics pipeline following PCR amplification of genetic material and subsequent sequencing of bacterial 16S rRNA.

**Results:**

Significant differences in both the alpha and beta diversities were found between the two groups (P<0.05). *Proteobacteria, Firmicutes*, and *Actinobacteriota* were identified as the predominant phyla in the placenta, while predominant identified genera included *Brevundimonas, Caulobacter, Lactobacillus*, and *Citrobacter*. There were no significant inter-group differences in the relative abundances of the predominant phyla and genera except *Lactobacillus*(P>0.05). Compared to infants without pneumonia, those with pneumonia demonstrated a decreased abundance of *Lactobacillus*, and an increased abundance of *Ureaplasma* and *Staphylococcus* (P<0.05). The relative abundance of *Ureaplasma* was positively correlated with that of *Staphylococcus*, and negatively correlation with that of *Lactobacillus* (P<0.05). Notably, we observed significant disparities in the metabolic pathways and phenotypes between the two groups (P<0.05).

**Conclusion:**

Overall, this study suggests that alterations in the placental microbiome may be linked to the onset of pneumonia in preterm infants. Further investigations are required to elucidate the relationship between microbiota and disease pathogenesis.

## 1 Introduction

Preterm birth, a common and serious complication of pathological pregnancies, is a significant contributor to neonatal mortality rates worldwide, exerting direct implications for population health outcomes (Bhandekar et al., 2024). Over one million premature births occur annually in China alone (Zhu et al., 2021). Advances in perinatal and neonatal care have led to noticeable improvements in survival rates among preterm infants (Wu et al., 2019); however, affected infants remain susceptible to various degrees of pulmonary injury, leading to heightened respiratory complications compared to their full-term counterparts (Huang et al., 2024). Pneumonia is an enduring consequence of premature birth, causing sustained lung damage, and jeopardizing the survival prospects of vulnerable newborns, consequently affecting their long-term quality of life. Management of preterm infants with pneumonia should include extended pulmonary rehabilitation (Wang et al., 2024). According to the life course theory, factors in various stages of human development can have long-term cumulative and interactive effects on both human development and disease. Changes in environmental factors during early life and pregnancy may also influence the occurrence of diseases in later life. The human microecosystem is defined as a complex network of the microbiotic communities and their habitats located within and on the body (Valentine et al., 2018). Evidence from prior studies has confirmed the presence of microorganisms in the placental tissue; however, it is important to note that these colonized organisms are not associated with perinatal infections (Piersigilli et al., 2020). Increasingly, studies have begun to characterize the dynamic microecosystem of pregnant women, which has been shown to undergo complex physiological changes closely linked to maternal and fetal health (Zheng et al., 2017a; La et al., 2022; Powell et al., 2024).

In recent years, a growing emphasis has been placed on the interplay between maternal, placental, and fetal microecologies. Vertical transmission of bacteria from the mother to fetus during pregnancy has also been scientifically validated, while the presence of symbiotic bacteria and lactic acid bacterial products in the meconium has been firmly established (Martin et al., 2016). The microbial colonization pattern in each neonate is unique, and subject to variability among individuals and anatomical locations (Wu et al., 2023). The human respiratory tract hosts a microbial community even before birth, while the airway microbiota of neonates delivered vaginally or via cesarean section are strikingly similar. It has thus been hypothesized that the fetal airway microbiota may have dual origins in both the placenta and amniotic fluid (Lal et al., 2016). However, research on the effects of placental microbiome on pneumonia in preterm infants remains limited.

To address this knowledge gap, the present study aimed to characterize and compare the placental microbiome of preterm infants with and without pneumonia.

## 2 Methods

### 2.1 Study design and setting

Preterm infants born between 32 and 36 weeks’ gestational age at the Chengdu Women and Children’s Central Hospital between June and December 2021 were enrolled in this study. The exclusion criteria included the offspring of mothers aged ≥ 35 years, received antibiotics during pregnancy, with a history of vaginitis or immune diseases during pregnancy, or pregnancies complicated by internal and surgical diseases. Additionally, infants with congenital developmental malformations (including complex congenital heart disease and pulmonary dysplasia), inherited metabolic diseases, chromosomal diseases, and those whose parents did not provide consent were also excluded. The indications for Cesarean section were based on an expert consensus on Cesarean section surgery published in 2014 (Obstetrics Group of the Chinese Association of Obstetrics and Gynecology, the Chinese Association of Obstetrics and Gynecology, and the Chinese Medical Association, 2014).

Apgar scores were determined by a qualified healthcare professional, in accordance with established protocols. An Apgar score of less than 7 was defined as low (American Academy of Pediatrics Committee on Fetus and Newborn and American College of Obstetricians and Gynecologists Committee on Obstetric Practice, 2015).Neonatal pneumonia was defined in accordance with the relevant clinical guidelines (Nissen, 2007).

The study protocol was approved by Chengdu Women and Children’s Central Hospital (2021 [123]). Written informed consent was obtained from the parents or guardians of the children who participated in this study. The study was conducted in accordance with The Code of Ethics of the World Medical Association (Declaration of Helsinki).

### 2.2 Sample collection

Immediately following preterm delivery, aseptic swabs were obtained by gently scraping the fetal surface of the placenta three times in sterile manner. Collected samples were promptly stored in a -80°C refrigerator within 30 minutes.

### 2.3 Bacterial DNA extraction

DNA extraction kits from Omega Bio-Tek (Madison, WI United) were used to ensure effective and high-quality DNA extraction. Following extraction, a target fragment library was constructed, sequencing PCR primers targeting conserved regions within the 16S rRNA gene were designed. Amplification of V3-V4 regions within the 16S rRNA gene was achieved through PCR utilizing the following primer pair: forward primer -341F (5’-CCTACGGGNGGCWGCAG-3’) and reverse primer -806R(5’-GACTACHVGGGTATCTAATCC-3’). Following thirty-five cycles of PCR amplification, sequencing adapters and barcodes were incorporated into the samples before detection by agarose gel electrophoresis at a concentration of 1.5%. Target fragments underwent recovery utilizing AxyPrep™PCR Cleanup Kits from Beckman Coulter Genomics based in Danvers Massachusetts, while purification involved employing the Quant-iT PicoGreen dsDNA Assay Kit from Invitrogen in America.

Additionally, Amplicon pools were prepared prior to assessment for quality assessment using both an Agilent 2100 Bioanalyzer and a Library Quantification Kit specifically designed for Illumina by Kapa Biosciences located in Woburn Massachusetts. The final step involved subjection of the library to sequencing on an Illumina NovaSeq PE250 platform, which resulted in the generation of paired-end reads measuring at 250 base pairs.

### 2.4 Bioinformatics pipeline

Paired-end reads were assigned to samples based on their unique barcodes, and were truncated by removing the barcode and primer sequences. Subsequently, the paired-end reads were merged using FLASH (V1.2.8). Quality filtering of the raw reads was conducted under specific conditions to obtain high-quality clean tags, following the QIIME (V1.9.1) quality control process. Chimeric sequences were filtered using Vsearch (V2.3.4). Effective tags were further obtained after dereplication using a divisive amplicon-denoising algorithm. Alpha and beta diversities were further calculated using QIIME2 (V.2019.7). An equal number of sequences was also randomly extracted by reducing the sequence count to match that of the sample with the fewest sequences, followed by calculation of the relative abundance (X bacterial count/total count) for bacterial taxonomy analysis. Images were generated using R software (V3.5.2). Species annotation sequence alignment was performed using BLAST with the SILVA and NT-16S alignment databases. Fisher’s exact test was applied to assess the differences in species at each taxonomic level.

### 2.5 Statistical analysis

Measurement data conforming to normal distribution are presented as x ± s, while non-normally distributed measurement data are presented as P50 (P25–P75). Comparisons between the two groups were conducted using the t-test and Mann-Whitney U test. The chi-square test was applied to compare count data. Spearman’s rank correlation analysis was used to examine the associations between different bacterial genera. All statistical tests were two-sided, and the results were considered statistically significant at P < 0.05. SAS 9.3 (SAS Institute Inc., Cary, NC, USA) was used for the data analysis.

## 3 Results

### 3.1 Clinical characteristics of the enrolled participants

A total of 59 preterm infants with gestational ages ranging from 32 to 35 weeks were enrolled in this study. Among them, 33 developed pneumonia within 48 hours of birth while the remaining 26 did not. The cohort comprised 40 male and 19 female infants (**Table 1**). There were 11 instances of spontaneous labor and 48 cesarean sections. Participants who developed pneumonia within 48 h after birth were assigned to Group A, whereas those who did not develop pneumonia were assigned to Group B. There were no significant differences in gestational age, 1-minute Apgar score, body weight, body length, chest circumference, or abdominal circumference between the two groups (P>0.05). However, the head circumference of infants in Group A was smaller than that of infants in Group B (P=0.026). Compared with Group B, Group A also tended to have longer hospital stay (30.24 + 17.98 days VS 9.04 + 6.0 days, P<0.001). There was no significant difference in the cesarean section rate between the two groups (84.45% VS 76.92%, P>0.05). However, in Group A, the most prevalent indications for cesarean section were fetal distress and severe preeclampsia, whereas in Group B, severe intrahepatic cholestasis of pregnancy and IVF-ET were the most prevalent indications. White blood cell counts within 24 hours after birth, neutrophils and eosinophils remained within the normal range. The difference in C-reactive protein between the two groups was statistically significant (1.0(1.0∼7.1)ng/ml VS 1.0(1.0∼1.0)ng/ml P < 0.05).

**Table 1:** Characteristics of the enrolled preterm infants with and without pneumonia.

	Pneumonia (n=33)	Control (n=26)	F/X^2^	P
Gender (F/M)	23/10	17/9	0.124	0.725
Gestational weeks	32.6 ± 1.98	34.62 ± 1.39	3.538	0.065
Weight (kg)	1.79 ± 0.46	2.27 ± 0.37	1.075	0.304
Length (cm)	40.32 ± 6.32	45.19 ± 2.06	3.443	0.069
Head circumference (cm)	29.23 + 2.17	31.27 + 1.47	5.257	0.026
Chest circumference (cm)	27.61 ± 2.32	29.77 ± 2.17	4.335	0.42
Abdomen circumference (cm)	26.15 ± 2.32	28.27 ± 1.79	1.615	0.209
Hospital stay (days)	30.24 ± 17.98	9.04 ± 6.0	15.37	<0.001
1 min Apgar score	8.15 ± 1.42	9.15 ± 0.78	3.118	0.083
Age of mother (y)	29.82 ± 4.46	29.8 ± 2.96	1.788	0.187
Age of father (y)	31.55 ± 3.92	32.52 ± 4.63	0.095	0.759
Delivery mode (natural/caesarean)	5/28	6/20	0.602	0.438
Cause of Caesarean section				
Chorioamnionitis	1	0	40.517	0
Chronic nephritis	2	0		
Premature rupture of fetal membranes	7	1		
Fetal distress in uterus	2	2		
Severe preeclampsia	7	4		
ICP	4	5		
Gestational hypertension	1	1		
Placental abruption	3	2		
IVF-ET	1	5		
Sputum culture *(Escherichia coli)*	1	0		
Blood culture *(Escherichia coli)*	2	0		
Antibiotics	25	0		
Respiratory support				
Mechanical ventilation	22	0		
CPAP	11	0		
WBC counts (*10^9^/L)	12.09 ± 9.35	11.12 ± 3.84	2.596	0.113
Neutrophils (%)	59.11 ± 18.56	52.05 ± 16.35	0.89	0.349
Eosinophils (%)	0.9 (0.25∼1.75)	1.95 (0.5∼3.5)	-2.514	0.012
CRP (ng/ml)	1.0 (1.0∼7.1)	1.0 (1.0∼1.0)	-2.318	0.017

CRP, C-reactive protein; ICP, intrahepatic cholestisis; IVF-ET, *in vitro* fertilization-embryo transfer; WBC, white blood cells.

### 3.2 Bacterial alpha diversity analysis

**Figure 1:**
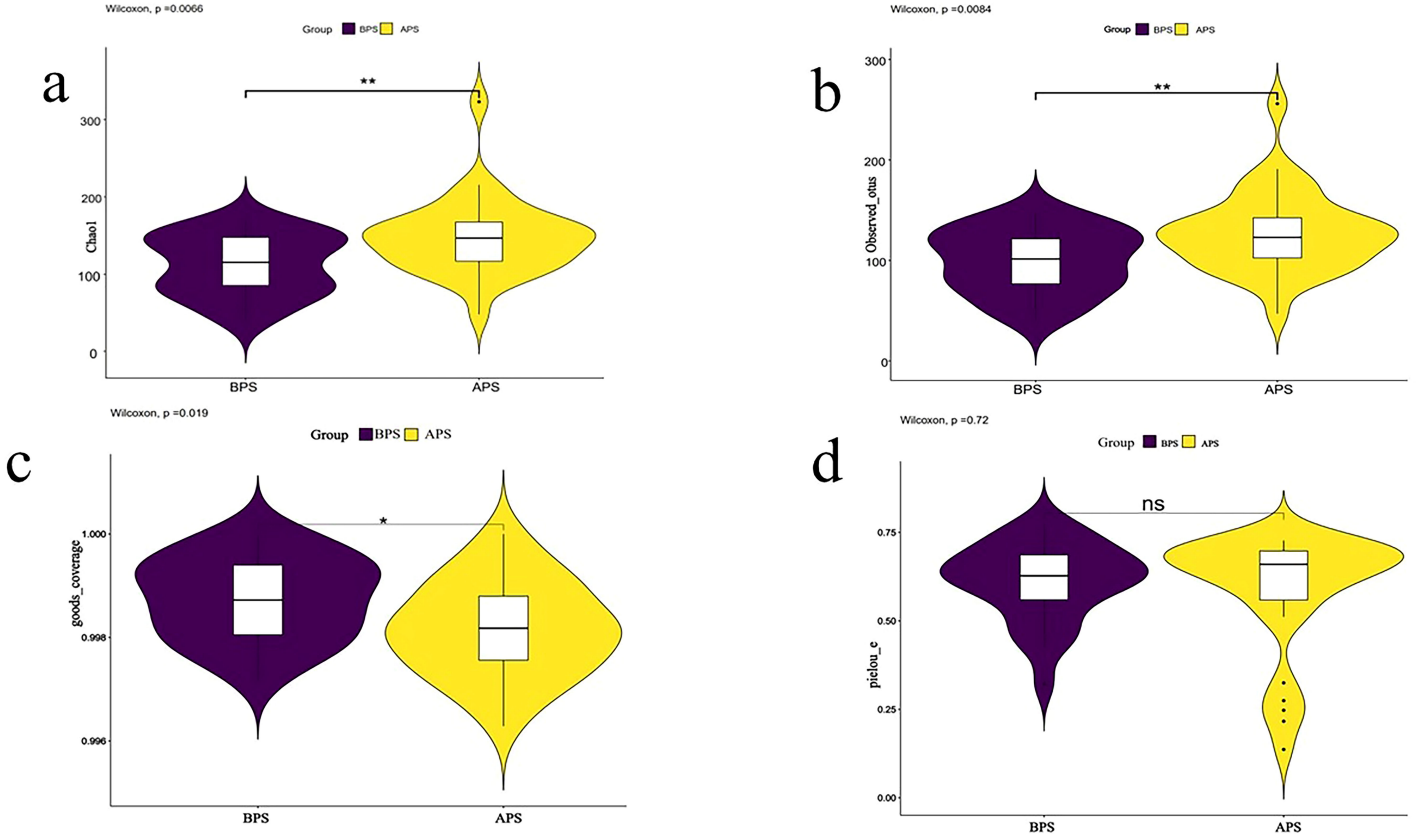
Results of alpha diversity analysis. Two groups of placenta swab 16S rRNA detection were divided into two groups: A placenta swab (APS) and B placenta swab (BPS). Alpha diversity is applied in analyzing complexity of species diversity for a sample through 4 indices, including **(A)** Chao1, **(B)**observed-species, **(C)** Goods_coverage, **(D)** pielou-e. Community richness can be assessed through the Chao1 and observed_species. Compared to BPS. *p<0.05; **p<0.01.

A higher richness of microbial populations was found in the APS (A placental swab) group (P<0.05) (**Figures 1A, B**). The sequencing depth exhibited statistically significant differences between the two groups, as measured by Good’s coverage (P<0.05, **Figure 1C**). The evenness of the species distribution determined by Pielou-e showed no significant differences between the two samples (P>0.05, **Figure 1D**).

### 3.3 Bacterial beta diversity analysis

**Figure 2:**
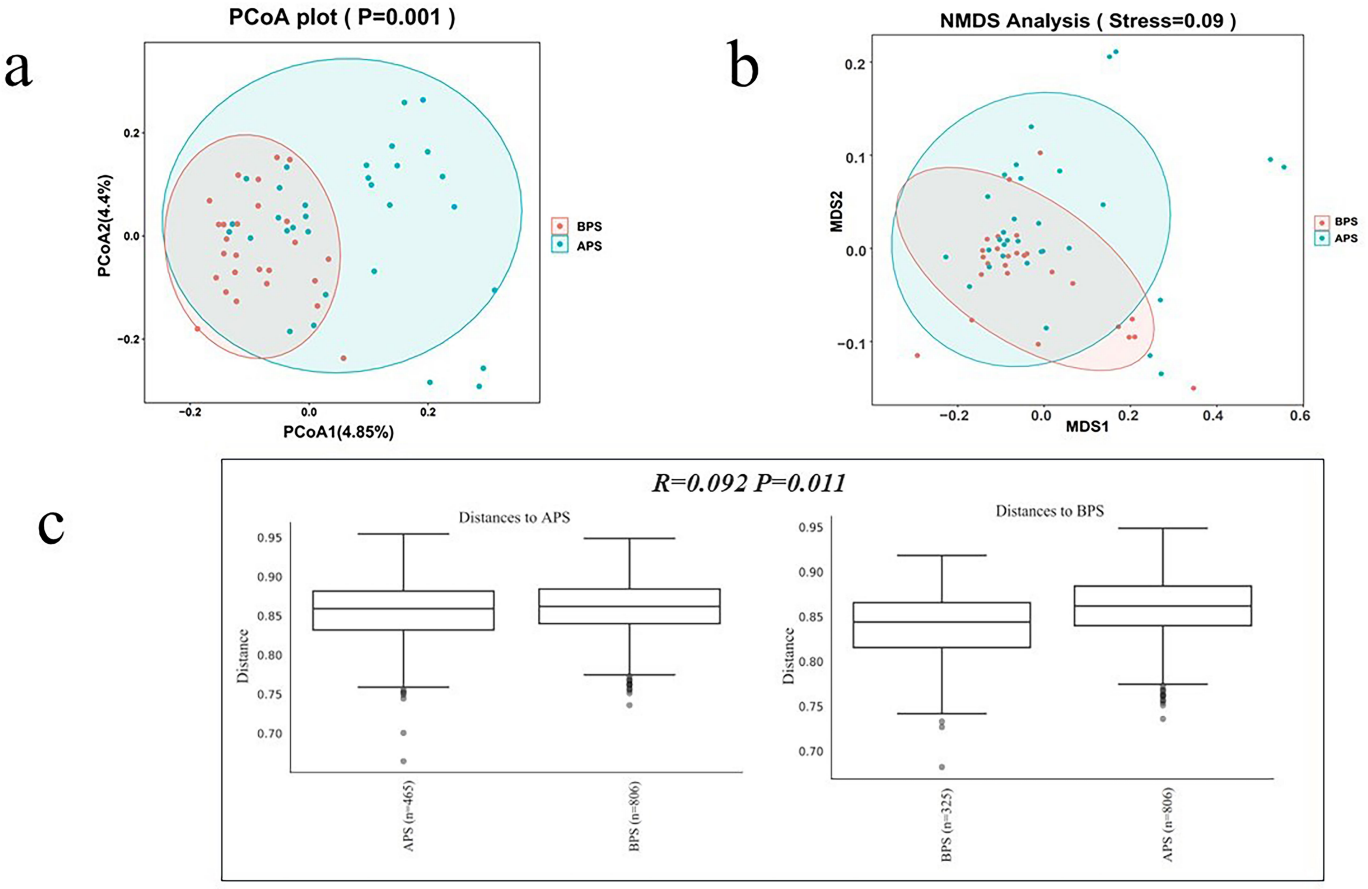
Bacterial beta diversity analysis. **(a)** PCoA analysis, **(b)** NMDS analysis, **(c)** ANOSIM analysis.

The ecological distances differed significantly between APS and BPS (B placental swab) by principal coordinate analysis (PCoA), as well as by nonmetric multidimensional scaling (NMDS) (P<0.05) (**Figures 2A, B**). Analysis of similarity (ANOSIM) is a non-parametric test widely applied to compare differences between groups with differences within groups. The ANOSIM results showed that the similarities within the groups were greater than those between the groups (P<0.05).

Beta diversity analysis was applied to evaluate the differences in species complexity. PCoA was performed to obtain the principal coordinates and to visualize complex multidimensional data. Non-metric multidimensional scaling (NMDS) is an indirect gradient analysis approach that produces an ordination based on a distance or dissimilarity matrix. The results of these analyses are presented in **Figure 2**. In **Figure 2A**, points of the same color denote they are from a common group, presented as a circle plot (≥4 biologic replicates per group), with 95% confidence intervals. Axis 1 represents the principal coordinate that explains the largest data change, while axis 2 represents the coordinate that accounts for the largest proportion of the remaining data changes. In **Figure 2B**, the spatial distance between sample points represents the difference between the samples. The axes were essentially arbitrary but displayed data in a manner that best represented their dissimilarity. Following the initial ordination, it was imperative to analyze the stress values generated by the algorithm. Stress values below 0.1 were considered to indicate good ordination with little risk of misinterpretation (**Figure 2B**). In **Figure 2C**, the test statistic R was bounded within the range of -1 to 1. Positive values indicate greater similarities within groups, whereas values equal to zero suggest no between-group differences or within-group similarities. Negative values suggest greater similarities between groups than within groups, potentially leading to incorrect sample assignments. In this study, the R-value was 0.092 (P<0.05).

### 3.4 Microbiota composition

**Figure 3:**
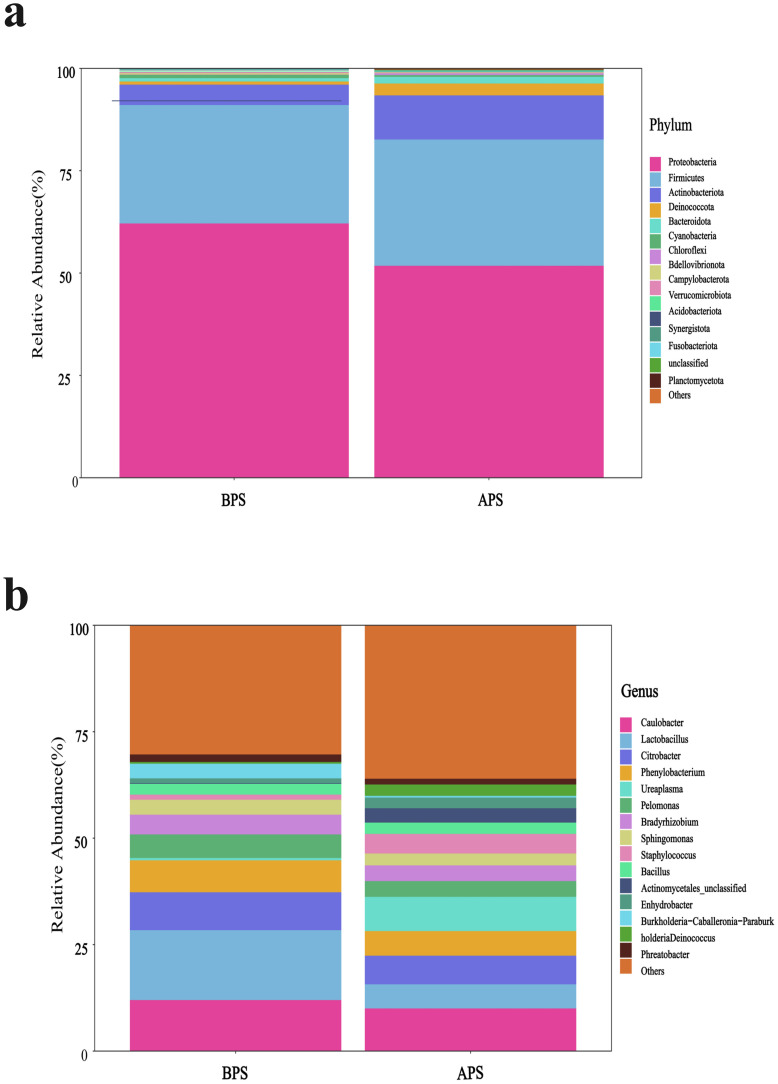
Microbiota composition, shown as the relative abundance plot of the top 15 species from placental swabs at the phylum **(A)** and genus levels **(B)**.

The microbiota of the APS and BPS were analyzed, encompassing a wide taxonomic range from phyla to genera. *Proteobacteria, Firmicutes*, and *Actinobacteriota* were the dominant phyla in both groups (**Figure 3A**), with discernible differences observed in dominant phyla between the two groups. At the genus level, the top five dominant genera in APS were *Brevundimonas*, *Caulobacter, Lactobacillus, Citrobacter*, and *Ureaplasma*, while those in the BPS were *Caulobacter, Lactobacillus, Citrobacter, Phenylobacterium*, and *Brevundimonas* (**Figure 3B**).

### 3.5 Taxa significant difference analysis

**Figure 4:**
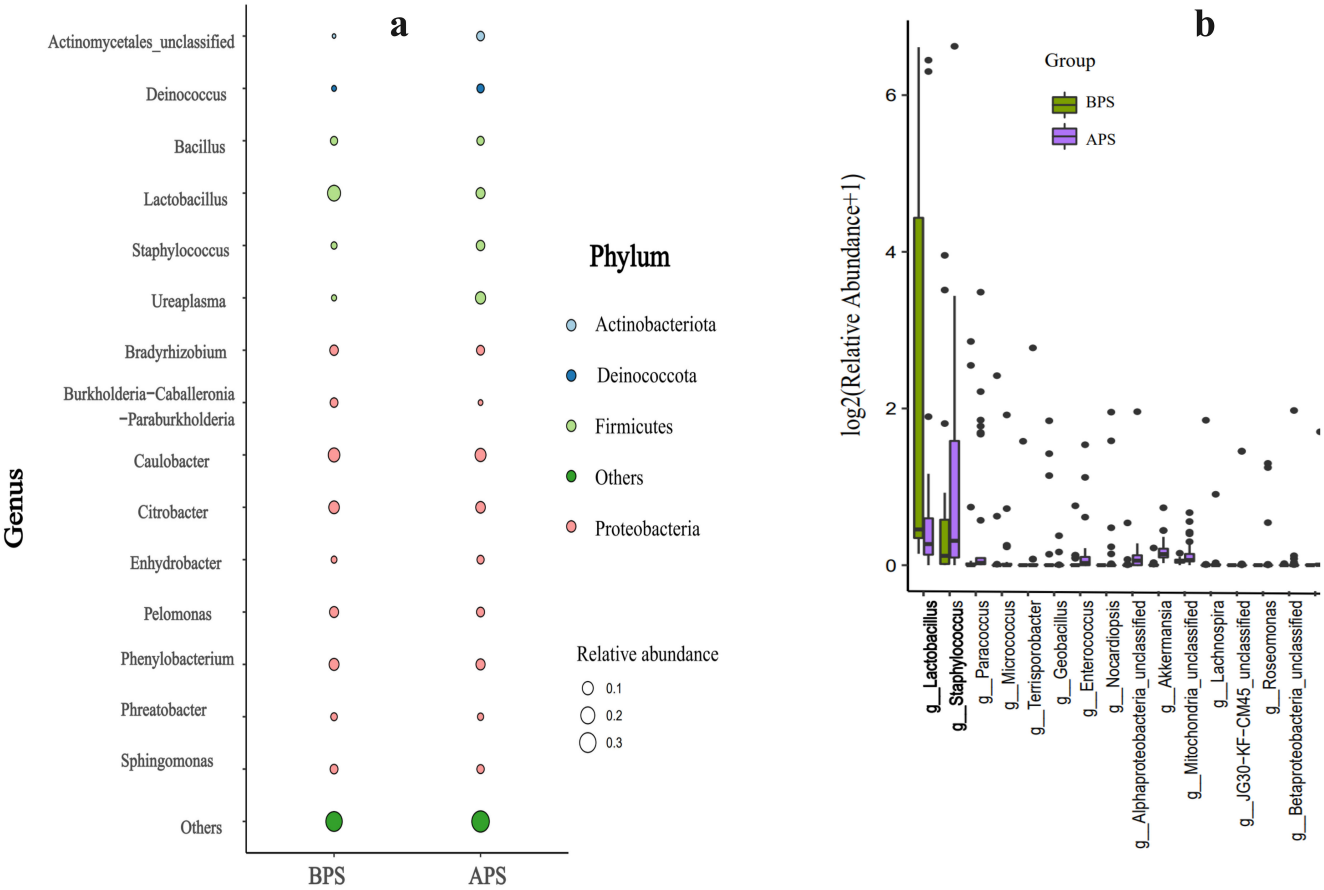
Results of microbiota significant difference analysis .Taxa significant difference analysis at the phylum **(a)** and genus **(b)** levels.

There were no significant inter-group differences in the relative abundances of the predominant phyla and genera except *Lactobacillus*(P>0.05).Compared to BPS, we observed a decrease in *Lactobacillus*, and an increase in *Ureaplasma* and *Staphylococcus* in APS, all of which belonged to *Firmicutes* (P<0.05) (**Figure 4A**). Fisher’s exact test was further applied to examine the disparity in species at the genus level, which revealed a significant reduction in *Lactobacillus* and a notable increase in *Staphylococcus* in APS(P < 0.05) (**Figure 4B**).

The distribution of taxa at the phylum and genus levels in APS and BPS is displayed in **Figure 4A**. Differences between APS and BPS at the genus level, and the top 15 species (P < 0.05) were selected for display in the bar chart in **Figure 4B**.

### 3.6 Correlation analysis between dominant genera

**Figure 5:**
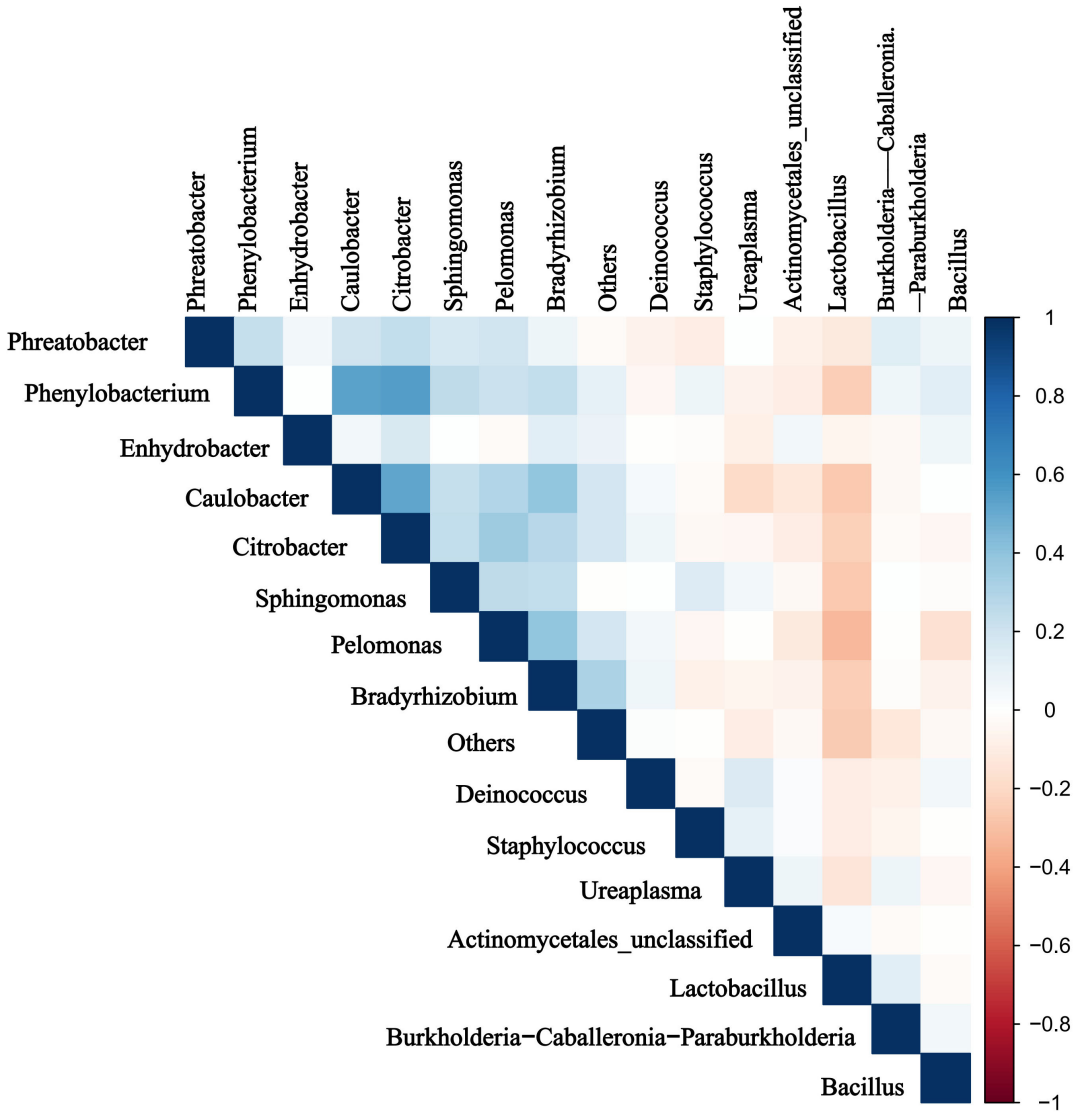
Results of the microbiota correlation analysis. Correlation heatmap of the top 15 dominant genera. Blue and red represent positive and negative correlations, respectively.

Significant rank correlations were observed among the dominant genera. Notably, relative abundance of *Ureaplasma* positively correlated with that of *Staphylococcus*, and negatively correlated with that of *Lactobacillus*(P < 0.05) (**Figure 5**).

### 3.7 Functional prediction and phenotypes analysis

**Figure 6:**
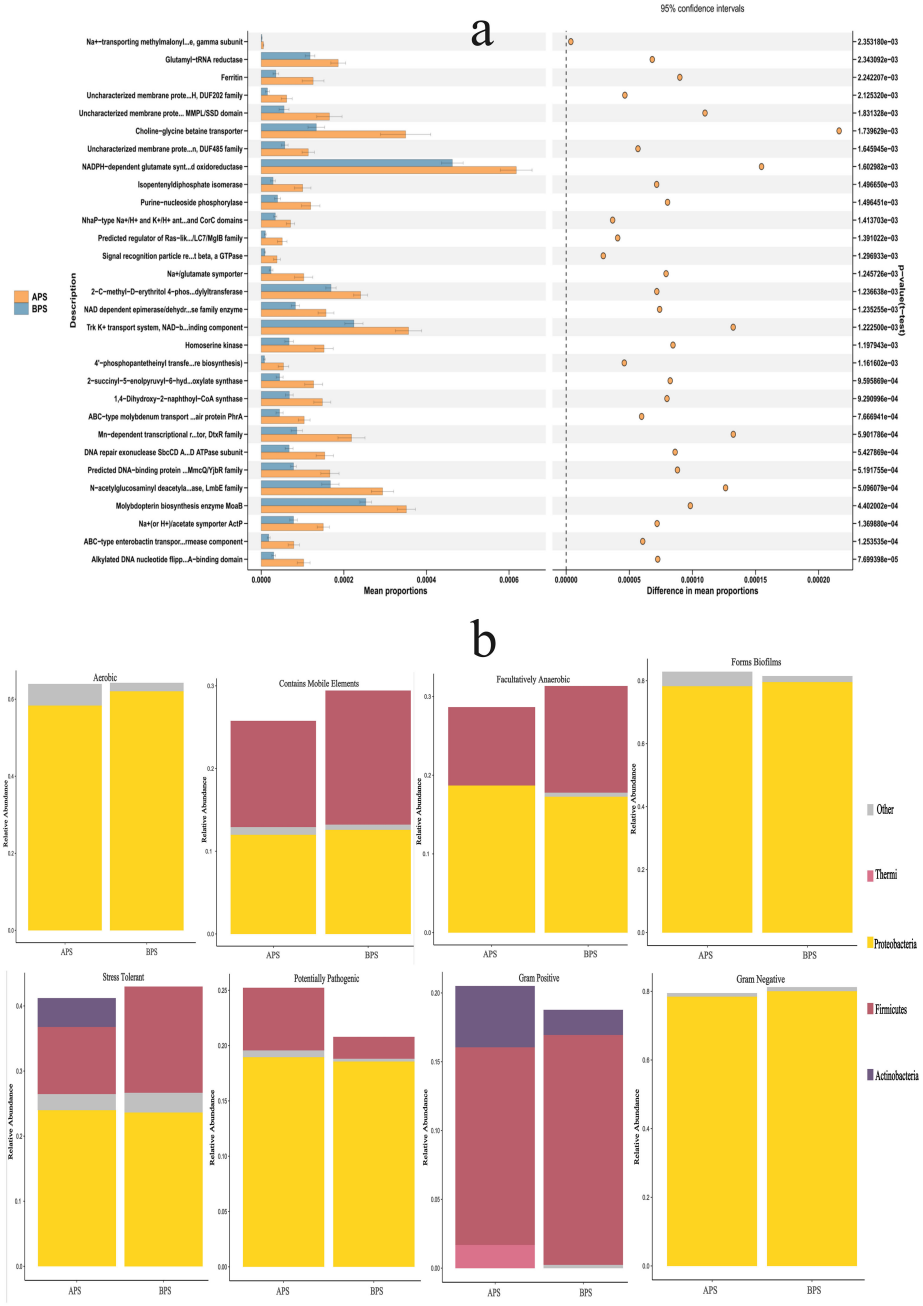
Results of functional prediction and phenotypes analysis. **(a)** Functional prediction of PICRUS, **(b)** Phenotypes analysis of BugBase.

Compared to BPS, the metabolic pathways related to glycogen degradation I (bacterial), the superpathway of menaquinol biosynthesis, the superpathway of hexitol degradation (bacteria), adenine and adenosine salvage, enterobactin biosynthesis, and glycogen degradation I (bacterial) were all found to be enriched in APS using PICRUS, based on the Clusters of Orthologous Genes (COG) database (**Figure 6A**). BugBase is a microbiome analysis tool that determines high-level phenotypes present in microbiome samples. Overall, there were nine identified phenotypes associated with aerobic, anaerobic, facultatively anaerobic, potentially pathogenic, stress tolerance, containing mobile element, biofilms formation, gram-negative bacteria and gram-positive bacteria. The dominant phenotypes in APS were aerobic, biofilms formation and gram negative at phylum level, so were in BPS. The relative abundances of the anaerobic and mobile element phenotypes were significantly different between the two groups (P<0.05) (**Figure 6B**).

The top 30 metabolic functions with a p-value <0.05 are shown in **Figure 6A**, while the relative abundances of phyla showing different phenotypes are presented in **Figure 6B**.

## 4 Discussion

In this study, the duration of hospital stay in group A was found to be prolonged, while infants diagnosed with pneumonia showed an elevated risk of requiring ventilator support. No significant differences were observed between the two groups in terms of gestational age, sex, birth weight, body length, or 1-minute Apgar score. The distribution of reasons for Cesarean section differed significantly between the two groups. The incidence of premature rupture of membranes and severe eclampsia increased in Group A, with the former identified as a risk factor for preterm birth. Neonatal infections and pneumonia have been shown to occur in infants born to mothers with chorioamnionitis (Jiang et al., 2018). One study investigating the bacterial communities in the placental tissues of 1391 women demonstrated that pregnant women with severe chorioamnionitis have an increased bacterial load and reduced species richness in the placenta (Prince et al., 2016). *Sneathia sanguinengens* and *Peptostreptococcus anaerobius* were also found to be associated with a smaller infant size. Further, another study observed dysbiosis of the gut microbiota in preeclamptic patients, which was characterized by significant reductions in the bacteria that produce short-chain fatty acids (Jin et al., 2022). Overall, these results suggest that gut microbiota dysbiosis plays an important role in preeclampsia development.

In the present study, the top three phyla identified in the placental microbiota of preterm infants were *Proteobacteria, Firmicutes*, and *Actinobacteriota*. This finding aligns closely with that of a previous study, which also identified *Proteobacteria*, *Actinobacteriota*, and *Bacteroidetes* as the predominant phyla (Aagaard et al., 2014). We hypothesized that the minor discrepancies between the two studies could be attributed to variations in ethnicity and regional factors. The relative abundances of the dominant phyla showed no significant inter-group differences. Further, *Brevundimonas*, *Caulobacter, Lactobacillus, Citrobacter* were all found to be prevalent at the genus level in both groups. These results differ from those of other studies (Seferovic et al., 2019; Zheng et al., 2017b; Benny et al., 2021), which may be explained by previous postulations that variations in the diseases under investigation contribute to disparities in placental microbiome composition. In the present study, we found that *Lactobacillus* were decreased, while *Ureaplasma* and *Staphylococcus* were increased significantly in APS, and that the abundances of *Ureaplasma* and *Staphylococcus* were negatively correlated with that of *Lactobacillus. Lactobacillus* confers numerous advantages to the human body, exerting antioxidative effects and antimicrobial activity, while promoting cholesterol metabolism, immunomodulation, anti-allergenic effects, and tumor suppression (Slattery et al., 2019). In one study, the researchers. compared vaginal samples and placental tissues from preterm and term mothers (Aslam et al., 2022), with results indicating that a decrease in *Lactobacillus* and an increase in *Ureaplasma* were correlated with preterm delivery. Dysbiosis of *Lactobacillus* and *Ureaplasma* in the placenta contributes to early onset pulmonary infections in preterm neonates. *Staphylococcus* is a prevalent bacterium in intrauterine infections and is a frequent causative agent of neonatal pneumonia (Mabena et al., 2024). In the present study, *Staphylococcus* was found to be more abundant in patients with APS. Farooqi et al. further explored the diversity of microbiota in full-term and preterm placental samples (Farooqi et al., 2022), finding that 16/68 (23.5%) full-term and 4/16 (25%) preterm placental samples were positive for *Staphylococcus aureus*. Nevertheless, the pathophysiological mechanisms underlying *Staphylococcus*-induced preterm birth remain unknown. As such, further research is required to prevent the transition of *Staphylococcus* from symbiotic microbes to pathogenic microorganisms and minimize its impact on neonates.

In the field of ecology, alpha diversity is a metric which represents the average species diversity within a specific site at a local scale, whereas beta diversity represents the ratio of regional to local species diversity. In the present study, we observed a significant difference in the alpha and beta diversities between the two groups. Some researchers have further noted that alterations in placental microbiota, which impact the outcome of delivery, are concomitant with changes in microbiota-mediated metabolic pathways (Prince et al., 2016; Aagaard et al., 2014; Antony et al., 2015). Antony et al. previously observed that the placentas of term-delivering women without chorioamnionitis exhibited a significant positive correlation with functional pathways related to the metabolism of various cofactors and vitamins, including riboflavin, nicotinate, pantothenate, and CoA (Antony et al., 2015). These pathways were similar to those of *Lactobacillus* in preterm women without chorioamnionitis. Virgiliou et al. previously investigated the metabolites in amniotic fluid samples taken from women who delivered preterm and at term, revealing significant differences in energy metabolism-related metabolites (pyruvic acid, glutamic acid, and glutamine) between these groups (Virgiliou et al., 2017). Our study similarly revealed variations in metabolic pathways and phenotypes across different diseases, suggesting that the placental microbiota may contribute to the development of the fetal innate immune system, and exert an impact on lung diseases in preterm infants. The transplacental mechanisms by which maternal taxonomy influences the offspring currently remains poorly understood (Piersigilli et al., 2020).

This study had several limitations. First, the sample size was relatively small. Therefore, large-scale studies are required. Secondly, the study sample did not include extremely preterm infants. As such, these findings are not representative of the entire population of preterm infants. Third, our study primarily focused on the quantification of bacterial abundance through 16S rRNA analysis, without delving into investigations pertaining to viruses and fungi. A comprehensive analysis of the bacterial, viral, and fungal microbiota, such as metagenomics could further enhance our understanding of host-microbiota interactions. Nevertheless, our results have the potential to significant contribute to the field of research.

## 5 Conclusion

Overall, this study represents the first attempt to investigate the impact of placental microecology on pulmonary diseases in preterm infants within this region. Throughout our investigation, we investigated the composition of the placental microbiota in preterm infants. Consequently, our research team aimed to conduct a comprehensive analysis of placental microecology and metabolomics across multiple regions with a large sample size to derive more precise conclusions regarding the influence of placental microecology on pulmonary infections in preterm infants. These results are important as they suggest that the placental microbiome may be linked to the onset of pneumonia in preterm infants, which could provide a basis for further research into novel methods to prevent this condition.

## Data Availability

The datasets presented in this study can be found in online repositories. The names of the repository/repositories and accession number(s) can be found in the article/supplementary material.
